# Relative Abundance and Detection of *Pseudomonas aeruginosa* from Chronic Wound Infections Globally

**DOI:** 10.3390/microorganisms11051210

**Published:** 2023-05-05

**Authors:** Sang Phan, Cafrey He Feng, Raymond Huang, Zeng X. Lee, Yer Moua, Olivia J. Phung, Justin R. Lenhard

**Affiliations:** College of Pharmacy, California Northstate University, Elk Grove, CA 95757, USA

**Keywords:** *Pseudomonas aeruginosa*, wound infection, diabetic foot infection

## Abstract

*Pseudomonas aeruginosa* is a difficult-to-treat pathogen that is frequently involved with chronic wound infections. Here, we conducted a literature search of world-wide studies published between 2005 and 2022 that described the microbiological profiles of chronic wound infections. For each continent, a hierarchy of pathogens was created to define the organisms that were most frequently isolated in each region. Except for South America, *P. aeruginosa* was the second most common organism in each major continent, with *Staphylococcus aureus* being the most abundant pathogen overall. When individual countries were evaluated, *P. aeruginosa* was the most frequently isolated organism in several Southeast Asia nations including India and Malaysia. *P. aeruginosa* was less commonly isolated from diabetic foot infections in North America, Europe, and Africa in comparison to other types of chronic wound infections. Additionally, the Levine wound swab technique may be a quick and painless way to isolate *P. aeruginosa* from wound infections, but the isolation of *P. aeruginosa* does not seem to be an informative predictor of the patient’s clinical course. A multivariate risk assessment that accounts for the regional frequency of *P. aeruginosa* isolation may be an appropriate way to guide empiric management of chronic wound infections.

## 1. Introduction

There are a variety of wounds that are vulnerable to infection from pathogenic bacteria. Acute wounds are often precipitated by an external breach of the patient’s skin and include lacerations, surgical incisions, burns, and traumatic injuries [1]. In contrast, chronic wounds are created by prolonged disruption to the barrier functionality of the patient’s skin, which is typically the result of comorbidities such as diabetes and peripheral vascular disease that compromise the maintenance and healing of dermal tissue. Diabetic foot infections and infected decubitus ulcers represent two of the most clinically relevant chronic wound infections. Although there are a diverse number of organisms that may cause chronic wound infections, polymicrobial infections often involve Gram-positive cocci, Gram-negative rods, and anaerobic bacteria [1,2,3]. Among the Gram-negative aerobes responsible for chronic wound infections, *Pseudomonas aeruginosa* is one of the most notorious pathogens due to the limited number of treatment options [4,5].

Despite being implicated in chronic wound infections, the significance of detecting *P. aeruginosa* from a patient’s infected wound remains controversial [6]. Some clinicians have hypothesized that *P. aeruginosa* may colonize the wound space and its eradication is not necessary for a chronic wound to heal, whereas a competing proposal states that *P. aeruginosa* is capable of severely damaging tissue and merits directed antibacterial therapy. In support of the latter belief, numerous studies have observed that *P. aeruginosa* virulence factors alter the ability of a wound to heal [7,8,9,10,11]. *P. aeruginosa* also possesses a complex relationship with other pathogens that cohabitate the same site of infection, and the organism may enhance the virulence of other pathogens [12,13], alter the pharmacodynamics of antibacterials directed at other organisms [14,15,16,17,18,19,20,21], and influence the structure of polymicrobial biofilms [13,22].

The optimal strategy for approaching pseudomonal wound infections is further complicated by both the difficulty of empirically determining if a chronic wound infection involves *P. aeruginosa* and also the ambiguity of interpreting clinical microbiology cultures obtained from wound swabs. High severity infections, exposure of lower extremities to water, and residence in a warmer region have all been posited as positive associations with the presence of *P. aeruginosa* in chronic wound infections [2,3,23]. In addition, the region of the world has been loosely tied to the likeliness of encountering *P. aeruginosa* in chronic wound infections, with the Eastern Hemisphere or Asia and Africa specifically being identified as areas with a high prevalence of *P. aeruginosa* [23,24]. When available, a deep tissue biopsy may increase the likeliness of detecting *P. aeruginosa* from a wound, but the invasive nature of obtaining deep tissue samples limits the technique to patients that are already receiving debridement or are at low risk for the spread of infection after tissue removal [1,2,3,23]. Although the clinical utility and proper technique for obtaining wound swabs are subjects of debate, many institutions continue to use wound swabs to inform antibacterial selection.

The current review seeks to analyze the recent literature to determine the relative abundance of *P. aeruginosa* in chronic wound infections based on the region of the world. The review also evaluates different techniques used to detect *P. aeruginosa* from chronic wound infections and assesses the associations between *P. aeruginosa* and clinical outcomes. The results of the analysis are intended to provide insight into clinical decision making regarding the management of chronic wound infections.

## 2. Methods

A literature search was conducted in PubMed to identify studies that evaluated the relative abundance of *P. aeruginosa* in chronic wound infections. The search terms “chronic wound microbiology” and “*pseudomonas* diabetic foot” were applied between January 2005 and December 2022. Additional studies were added at the discretion of the authors. References were included in this review if they were studies conducted in human subjects and reported values related to the relative abundance of microorganisms isolated from chronic wound infections. Studies that reported on the techniques used to isolate *P. aeruginosa* from chronic wound infections and investigations that evaluated risk factors for *P. aeruginosa* involvement with wound infections were also included. Based on the study, *P. aeruginosa* detection may have been mediated through a variety of wound culturing techniques or through rapid diagnostics such as PCR. Data were extracted in a tabular format and qualitatively assessed.

The ranking of the most commonly encountered microorganisms in each continent and country represented by at least one reference was determined by collating the relative abundance of the pathogens reported in all the studies conducted in the region. For example, if a certain microorganism was the most abundant organism in the majority of the studies in a specific geographic region, it was determined to be most abundant for that region, and so on. The relative abundance of each pathogen was also determined for the subset of studies that investigated diabetic foot infections specifically.

In addition to determining the relative abundance of each pathogen, the frequency of *P. aeruginosa* isolation was estimated for each continent. Depending on the study, the frequency of *P. aeruginosa* isolation may have been reported based on the total number of patients, the number of positive cultures, or the number of total isolates. Due to the variability with how study results were reported, the frequency of isolation reported by the authors was used for each study in our analysis and readers are referred to individual studies for specific reporting information. If a study only reported on *Pseudomonas* species, then *P. aeruginosa* was assumed to be the dominant specie.

## 3. Prevalence of *P. aeruginosa* in Wound Infections by Region of the World

### 3.1. North America

**Figure 1:**
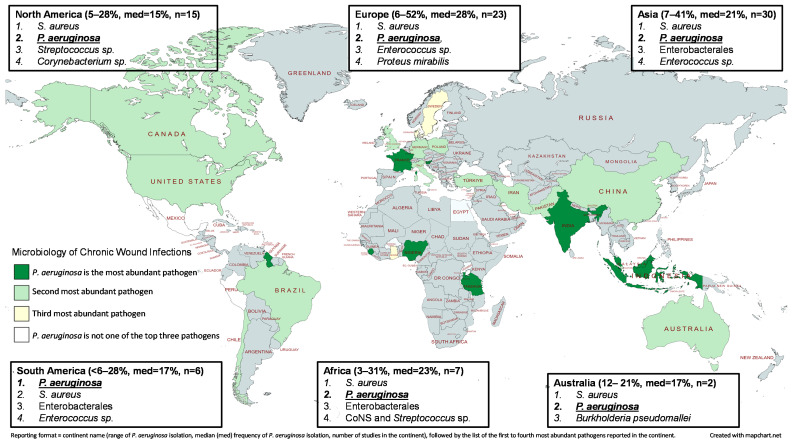
Visual depiction of the relative abundance of *P. aeruginosa* in chronic wound infections of any type across the entire world. Each country that is represented by at least one study is shaded as dark green (*P. aeruginosa* was the most abundant pathogen), light green (*P. aeruginosa* was the second most abundant pathogen), yellow (*P. aeruginosa* was the third most abundant pathogen), or white (*P. aeruginosa* was not one of the top three pathogens isolated from chronic wound infections). In addition, the comparative abundance of organisms within a continent are listed along with the range and median (med) percentage of how frequently *P. aeruginosa* was isolated as reported in each study.

There were 15 studies identified from North America that described the microbiology of chronic wound infections (Figure 1) [25,26,27,28,29,30,31,32,33,34,35,36,37,38,39]. The majority of the studies corresponded to patient populations within the United States of America, and the most commonly isolated pathogen across the entire continent was *S. aureus*. *P. aeruginosa* was the second most commonly isolated organism, with a range of detection of 4.5–28% (median ~15%). Only four studies were conducted in the outpatient setting, whereas the majority of the investigations focused on inpatients. The investigation by Wolcott et al. included the largest isolate collection, which was obtained from 2963 patients with chronic wounds [33]. In the study, *P. aeruginosa* was detected in 25% of samples and was the second most prevalent pathogen behind *S. aureus*. The researchers also observed that *P. aeruginosa* was relatively more abundant in venous leg ulcers and decubitus ulcers in comparison to diabetic foot ulcers and nonhealing surgical wounds.

**Figure 2:**
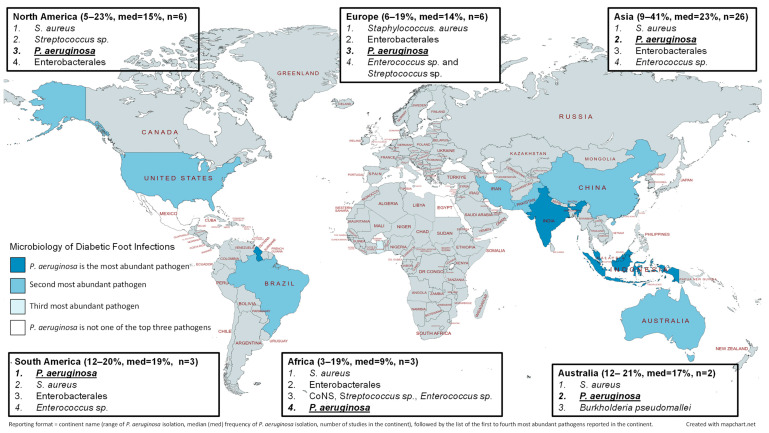
Relative abundance of *P. aeruginosa* in comparison to other pathogens that were isolated in studies that focused on diabetic foot infections specifically. Each country that is represented by at least one study is shaded as dark blue (*P. aeruginosa* was the most abundant pathogen), light blue (*P. aeruginosa* was the second most abundant pathogen), slight tint of blue (*P. aeruginosa* was the third most abundant pathogen), or white (*P. aeruginosa* was not one of the top three pathogens isolated from chronic wound infections). In addition, the comparative abundance of organisms within a continent are listed along with the range and median (med) percentage of how frequently *P. aeruginosa* was isolated as reported in each study.

Of the 15 studies that corresponded to wound infections, six studies focused exclusively on patients with diabetic foot infections (Figure 2) [34,35,36,37,38,39]. Five studies evaluated patients from the United States of America and had a median detection of *P. aeruginosa* of 14.5%, whereas the single study from Mexico observed *P. aeruginosa* involvement with only 7% of infections. Overall, *P. aeruginosa* was the third most prevalent organism in North American diabetic foot infections, but the pathogen was still the most commonly encountered gram-negative organism. The largest evaluation of diabetic foot infections was conducted by Henig et al. who assessed patients who were admitted to the Detroit Medical Center in the United States of America [35]. The definition of multidrug-resistant organisms (MDROs) used by the study authors included specific drug-resistant phenotypes of certain pathogenic bacteria, as well as any antimicrobial susceptibility profile of *P. aeruginosa*, *Acinetobacter baumanii*, and *Stenotrophomonas maltophilia*. Of the 648 patients included in the study, MDROs were detected in 346 patients. The second most common MDRO was *P. aeruginosa* (behind methicillin-resistant *S. aureus*), which was detected in 26% of patients with MDROs. *P. aeruginosa* was also the most common MDRO recovered from polymicrobial infections.

### 3.2. South America

Three studies from Brazil [40,41,42], one analysis from Chile [43], one investigation from Guyana [44], and an evaluation from Peru [45] were retrieved in the literature search for a total of six South American wound infection studies (Figure 1). Although *S. aureus* and *P. aeruginosa* were separately identified as the most commonly isolated organisms in two studies each, *P. aeruginosa* was more frequently reported as the second most abundant pathogen, which resulted in *P. aeruginosa* being the most prevalent organism in South American wound infections in the current review. The lowest rate of *P. aeruginosa* isolation was observed in a Peruvian study that observed 6% of 75 chronic wound infections involved either *Pseudomonas* or *Acinetobacter* species [45]. In contrast, a Brazilian investigation of chronic leg ulcerations isolated *P. aeruginosa* from 29% of the patients with infected ulcers [40]. The authors also observed that *P. aeruginosa* was the most common organism that was present in non-infected ulcerations. When all South American studies were aggregated, the median rate of *P. aeruginosa* isolation was ~17%.

Two studies in Brazil and the investigation in Guyana evaluated diabetic foot infections specifically (Figure 2) [41,42,44]. In the three studies, the rate of *P. aeruginosa* isolation from wound infections were 12%, 18.8%, and 19.6%, respectively. The largest study was conducted by Cardoso et al. who observed that *P. aeruginosa* was the most commonly isolated organism from 189 diabetic foot infections with a frequency of isolation of 19.6% [42]. *P. aeruginosa* was also the most commonly isolated organism in a Guyanese study that evaluated 183 diabetic foot infections [44], whereas *P. aeruginosa* was the fourth most common pathogen in a Brazilian evaluation of 99 patients with diabetic foot infections [41].

### 3.3. Europe

A total of 23 studies were identified that described the microbiology of chronic wound infections in Europe. Germany contributed four studies [46,47,48,49], and three separate studies were conducted within Denmark [50,51,52], Italy [53,54,55], the United Kingdom [56,57,58], Poland [59,60,61], and Turkey [62,63,64]. France [65], Portugal [66], the Republic of Slovenia [67], and Sweden [68] each contributed a single investigation. *S. aureus* was the most frequently encountered pathogen in Europe followed by *P. aeruginosa*, which was the most prevalent organism in three studies [61,65,67] and the second most abundant pathogen in the majority of the remaining studies. The only studies that corresponded to France and Slovenia reported *P. aeruginosa* as the common organism isolated from wound infections [65,67]. A study by Kwiecińska-Piróg et al. evaluated a large collection of 1142 bacterial cultures from wound infections in Poland and found that *P. aeruginosa* was the second most common pathogen and was associated with 35% of the positive wound cultures [59]. In addition, 75% of the wound infections were polymicrobial, and the two most common bacterial duos were *P. aeruginosa* cultured with either *S. aureus* or *P. mirabilis*. Overall, the median rate of *P. aeruginosa* isolation was 23% in Europe.

Six European studies focused specifically on diabetic foot infections. Half of the investigations took place in Turkey [62,63,64], whereas two studies were conducted within the United Kingdom [56,57] and a single study was included from Portugal [66]. The three studies from Turkey all reported that *P. aeruginosa* was the second or third most common pathogen in diabetic foot infections with a prevalence of *P. aeruginosa* that ranged from 17.3–19% [62,63,64]. The prevalence of *P. aeruginosa* in the two studies from the United Kingdom was 6.1% and 8.6% [56,57], respectively, whereas *P. aeruginosa* was present in 11.4% of the wound cultures evaluated in Portugal [66].

### 3.4. Asia

There were 30 studies conducted in Asian countries that discussed the microbiology of wound infections and the prevalence of *P. aeruginosa* (Figure 1). Countries that were represented in the literature search included China [69,70,71], India [72,73,74,75,76,77,78,79,80,81,82], Indonesia [83,84], Iran [85,86], Kuwait [87], Lebanon [88], Malaysia [89,90,91], Pakistan [92,93,94], Singapore [95], Turkey [96], and Iraq [97]. Most of the included studies focused on diabetic foot infections, and the frequency of *P. aeruginosa* isolation was therefore comparable when all chronic wound infections were evaluated or if diabetic foot infections specifically were analyzed (Figure 1 and Figure 2). Across the entire continent, *S. aureus* was the most commonly encountered organism, while *P. aeruginosa* was typically the second most common pathogen with a rate of isolation of about 21% (Figure 1).

Contrary to the rest of the continent, *P. aeruginosa* was the most abundant pathogen in India and Malaysia. Of the 11 studies that took place in India, five of the investigations found that *P. aeruginosa* was the most commonly isolated organism, which was followed by *S. aureus* and members of the order Enterobacterales (mainly *Escherichia coli* and *Klebsiella pneumoniae*), respectively [72,73,74,75,76,77,78,79,80,81,82]. The reported frequency of *P. aeruginosa* isolation was also relatively consistent with a range of 16–30% in every Indian study. *P. aeruginosa* was also the most abundant pathogen in Malaysia, where two of three studies reported that *P. aeruginosa* was the most frequently encountered organism [89,90,91]. In Indonesia, *P. aeruginosa* and *S. aureus* were both reported as the most abundant organism in one study and the second most common organism in the other investigation conducted in the country [83,84].

### 3.5. Africa

Seven studies that evaluated the microbiology of chronic wound infections in Africa were included in the review (Figure 1). A single investigation was identified from each of the following countries: Ghana [98], Tanzania [99], Burkina Faso [100], Sierra Leone [101], Nigeria [102], Uganda [103], and Egypt [104]. Similar to other regions, *S. aureus* was the most frequently encountered pathogen in the continent of Africa, and *P. aeruginosa* was the second most abundant organism; however, *P. aeruginosa* was the most commonly isolated organism in the studies that corresponded to Nigeria, Sierra Leone, and Tanzania [99,101,102]. The lowest rate of *P. aeruginosa* isolation (2.8%) was observed in Burkina Faso [100], whereas the Nigerian investigation reported the highest *P. aeruginosa* rate of isolation (30.6%) [102]. The median frequency of *P. aeruginosa* isolation for the entire region was 23%.

The investigations from Egypt, Ghana, and Burkina Faso focused specifically on diabetic foot infections [98,100,104]. Collectively, *P. aeruginosa* was a relatively rare organism to isolate from diabetic foot infections with an approximate isolation rate of 9.4%. Not only were gram-positive cocci more commonly encountered in African diabetic foot infections, but members of the order Enterobacterales were reportedly the most prominent gram-negative pathogens. The largest study in the African region evaluated 1803 isolates from diabetic foot infections in Cairo, Egypt, and found that *P. aeruginosa* was the third most encountered organism and the most prevalent gram-negative pathogen [104]. The Egyptian investigation also observed that *P. aeruginosa* was more commonly cultured from outpatients with diabetic foot infections in comparison to inpatients.

### 3.6. Australia

Only two Australian studies that discussed the microbiology of chronic wound infections were located in the literature search and both focused on diabetic foot infections (Figure 1 and Figure 2) [105,106]. An analysis by Lynar et al. reviewed a collection of microbiological samples that were taken from 413 adult patients living with diabetes and found that *P. aeruginosa* was isolated from 12.2% of the patients’ samples [106]. In a study by Commons et al., *P. aeruginosa* was detected in 20.9% of diabetic foot infections that resulted in hospital admissions during the study period [105]. When the authors included wound samples from the 12 months prior to hospital admission, then *P. aeruginosa* was isolated from 26.6% of the 177 patients that were included in the analysis. In both of the aforementioned studies, *S. aureus* was the most commonly isolated pathogen and *P. aeruginosa* was the second most commonly reported organism; however, only a few pathogens were discussed in each study.

## 4. Other Risk Factors for Involvement of *P. aeruginosa* in Chronic Wound Infections

To prevent the unnecessary use of broad spectrum antimicrobials for the treatment of chronic wound infections, it is helpful to empirically assess the likelihood that *P. aeruginosa* is involved with a given infectious process. As outlined in the preceding section, the geographic region and type of chronic wound infection may both influence the probability of encountering *P. aeruginosa*. Moreover, two separate studies found that large wounds with average sizes of 35.89 cm^2^ and 42.8 cm^2^ were correlated with the presence of *P. aeruginosa* (*p* = 0.0014 and *p* < 0.001, respectively) [46,50]. The isolation of *P. aeruginosa* was also associated with longer wound durations (*p* < 0.0001) [46], prior amputations (*p* < 0.001) [107], and the use of an active wound dressing in the past (*p* = 0.018) [107]. In contrast, the severity of the wound infection, recent antimicrobial use, and the presence of osteomyelitis were not associated with *P. aeruginosa* in two separate analyses [39,100].

## 5. Clinical Predictive Value of Culturing *P. aeruginosa*

In patients suffering from diabetic foot infections, multiple studies failed to establish a statistical relationship between the presence of *P. aeruginosa* and the likeliness that the patient will require an amputation or surgical debridement [39,41,42]. The involvement of *P. aeruginosa* was also not associated with whether patients requiring surgical interventions will receive minor versus major amputations (*p* > 0.05) [107]. A multivariable analysis of diabetic foot infections by Saltoglu et al. determined that culturing gram-negative rods was associated with an increased risk of limb loss (*p* = 0.022), and although *P. aeruginosa* was the most abundant gram-negative rod, there were approximately twice as many Enterobacterale isolates collectively, which confounds the interpretation of the study [64]. According to an investigation by Zhang *et al*., the relationship between *P. aeruginosa* and the likeliness of amputations for patients with diabetic foot infections may depend on the specific toxins that are released by each strain of *P. aeruginosa* [70].

In addition to a lack of an association between *P. aeruginosa* and amputations, the presence of *P. aeruginosa* has failed to correlate with other clinically meaningful processes as well. Despite being associated with the production of biofilm in wound infections [55,81], *P. aeruginosa* was not associated with delayed wound healing in chronic leg ulcerations [58]. In a separate investigation, the lack of *Pseudomonas* species being detected from leg ulcerations was associated with delayed wound healing in a multivariate analysis (*p* = 0.005) [108], but the specific impact of *P. aeruginosa* on wound healing may depend on the virulence factors possessed by each individual strain [70]. Another study found that *P. aeruginosa* was not associated with rehospitalization (*p* = 0.541) or mortality (*p* = 0.374) [62], and while a second investigation determined that *P. aeruginosa* was associated with mortality in a univariate analysis, the multivariate model removed *P. aeruginosa* as an independent predictor of mortality (*p* = 0.16) [106]. *P. aeruginosa* was also associated with necrotizing wound infections in a separate univariate analysis (*p* = 0.004), but again, the multivariate analysis did not include *P. aeruginosa* as a predictor of necrotizing infections [109].

## 6. Sampling Techniques for the Detection of *P. aeruginosa*

As discussed earlier, the optimal technique for isolating organisms from chronic wound infections is a subject of debate [1]. Although a tissue biopsy may decrease the likeliness of culturing superficial colonizers, the technique has several drawbacks including cost, time, and patient pain and bleeding. If wound swabs are used, clinicians may elect to either use a Z technique that involves manipulating the swab in a zig-zag formation over the center of the wound, or conversely, the Levine technique requires the medical professional to rotate a sterile swab over a 1 cm^2^ section of clean wound tissue with enough pressure to expel fluid from the wound [110,111].

To determine the optimal way to specifically detect *P. aeruginosa* from chronic wound infections, clinicians must first know whether tissue biopsies and wound swabs result in different rates of detection. An analysis by Davies et al. suggested that wound swabs were generally equivalent to punch biopsies for the detection of pathogens from chronic leg ulcerations, but a subanalysis of *P. aeruginosa* was not available [58]. A study by Gjødsbøl et al. evaluated sample collection techniques of chronic venous leg ulcers and determined that tissue biopsies and wound swabs resulted in similar microbiological profiles, and there was no statistical difference in the isolation of *P. aeruginosa* (*p* = 0.90) [51]. When Smith et al. compared curetted tissue samples and wound swabs of uninfected chronic wounds of patients that use intravenous drugs, wound swabs cultured a greater yield of anaerobic and gram-positive bacteria; however, both techniques resulted in the isolation of *P. aeruginosa* from the same number of patients [32].

Perhaps the largest study that compared wound swabs with tissue samples was a multicenter, prospective, cross-sectional study conducted by Nelson et al. that evaluated diabetic foot infections [57]. Over 400 patients were included in the analysis, and the authors found that tissue samples detected more pathogens than Levine’s technique (*p* < 0.01), but the tissue samples obtained from a curette or scalpel resulted in more ulcer pain and bleeding. Both sampling techniques detected *P. aeruginosa* in a total of 26 patients, with 18 patients that overlapped between the two groups. The overall rate of agreement between the two techniques was 95.9% (prevalence-adjusted kappa 0.92) for *P. aeruginosa*.

Given the similar rates of *P. aeruginosa* isolation when a wound swab or tissue sample is used, it may be helpful to determine if different wound swab techniques impact the likeliness of culturing *P. aeruginosa* [28]. A study by Gardner et al. compared tissue culture results from chronic wound infections with results obtained from Levine’s technique, the Z technique, and wound exudates. Not only was the Levine technique superior to the Z technique and wound exudates overall, but the Levine technique also had the highest level of agreement with tissue culture results when *P. aeruginosa* was specifically evaluated. In a randomized controlled trial that compared the Levine technique to the Z technique for the isolation of pathogens from acute and chronic wounds, the Levine technique was superior to the Z technique for collection of organisms from both acute and chronic wounds [110]. Both techniques were able to detect *P. aeruginosa*, but a quantitative analysis for *P. aeruginosa* specifically was not available. Based on the results of the aforementioned studies, the Levine technique may be one of the most pragmatic and reliable ways to isolate *P. aeruginosa* from a chronic wound infection.

## 7. Limitations and Areas for Further Research

There are several limitations to the current review that are relevant to the interpretation of the information presented within the manuscript. First, our global summary of the relative abundance of *P. aeruginosa* in chronic wound infections is only intended to give an approximation of how commonly *P. aeruginosa* is encountered in a certain area in comparison to other pathogens. Given the heterogeneity of the studies that were included in the review, it was difficult to make objective comparisons between different regions. Many of the included investigations used different patient populations, evaluated different types of chronic wound infections, used diverging study designs, or reported their results in contrasting manners. Second, our review was restricted to countries that published studies relevant to the current subject, and given the different size and geographical characteristics of every country, there may be important differences in the microbiological distribution of pathogens within a country that were not addressed [46]. Lastly, as we addressed in Section 4 of the manuscript, geography is just one variable that influences the likeliness of *P. aeruginosa* participation in a chronic wound infection, and clinicians must use a multifactorial decision-making process when determining if *P. aeruginosa* is a suspected pathogen.

Based on the results of our review, there are several areas of potential exploration that will benefit from additional research. The global distribution of studies we located were asymmetrically clustered in certain regions of the world, and areas without a local study that clarifies the regional distribution of microorganisms in a chronic wound infection will likely benefit by conducting such an investigation. Eventually, it will be helpful if the healthcare community can develop a validated screening system that accounts for the region and patient-specific characteristics to prospectively assess the likeliness that *P. aeruginosa* and other difficult-to-treat pathogens are involved in a chronic wound infection.

## 8. Conclusions

In summary, the studies included in the current review suggest that *P. aeruginosa* is the second most common pathogen isolated from chronic wound infections globally. The relative abundance of *P. aeruginosa* varied based on the type of wound infection and among countries within a single continent. India, Malaysia, and other nations in Southeast Asia may consider having a lower threshold for suspicion of *P. aeruginosa* involvement with chronic wound infections given the high abundance of the organism in that region. We were not able to locate definitive evidence that tissue samples are more likely to detect the presence of *P. aeruginosa* in comparison to the less invasive Levine swab technique, and in general, the presence of *P. aeruginosa* did not seem to correlate with clinical outcomes such as the likeliness of limb amputation or mortality. Considering that risk factors such as wound size and duration of the wound were associated with *P. aeruginosa* isolation, clinicians may be able to develop risk stratification systems that incorporates region and other variables into an empiric plan for the management of each wound infection.
